# The stress response gene *ATF3* is a direct target of the Wnt/β-catenin pathway and inhibits the invasion and migration of HCT116 human colorectal cancer cells

**DOI:** 10.1371/journal.pone.0194160

**Published:** 2018-07-02

**Authors:** Makoto Inoue, Yohei Uchida, Makoto Edagawa, Manabu Hirata, Jun Mitamura, Daiki Miyamoto, Kenji Taketani, Shigeki Sekine, Junya Kawauchi, Shigetaka Kitajima

**Affiliations:** 1 Department of Biochemical Genetics, Medical Research Institute, Tokyo Medical and Dental University, Tokyo, Japan; 2 Department of Surgery and Sciences, Graduate School of Medical Sciences, Kyushu University, Fukuoka, Japan; 3 Pathology Division, National Cancer Center Research Institute, Tokyo, Japan; University of Kentucky, UNITED STATES

## Abstract

Aberrant Wnt/β-catenin signaling is implicated in tumorigenesis and the progression of human colorectal cancers, and mutations in the components of the Wnt/β-catenin signaling pathway are observed in the majority of patients. Therefore, extensive studies on the Wnt signaling pathway and its target genes are crucial to understand the molecular events of tumorigenesis and develop an efficacious therapy. In this study, we showed that the stress response gene *ATF3* is transcriptionally activated by the binding of β-catenin and TCF4 to the redundant TCF4 site at the proximal promoter region of the *ATF3* gene, indicating that *ATF3* is a direct target of the Wnt/β-catenin pathway. The loss of function or overexpression studies showed that ATF3 inhibited the migration or invasion of HCT116 cells. The expression of some *MMP* and *TIMP* genes and the ratio of *MMP2/9* to *TIMP3/4* mRNAs was differentially regulated by ATF3. Therefore, though *ATF3* is activated downstream of the Wnt/β-catenin pathway, it acts as a negative regulator of the migration and invasion of HCT116 human colon cancer cells exhibiting aberrant Wnt/β-catenin activity. ATF3 is a candidate biomarker and target for human colorectal cancer treatment and prevention.

## Introduction

The Wnt signaling pathway, which is conserved in various species, determines cell fate and tissue development [1]. It consists of two different major pathways, namely, the canonical and the non-canonical pathways [2]. The activation of the canonical Wnt signaling pathway is triggered by the binding of Wnt molecules to the Frizzled receptors (Fz) [3] and LDL-receptor related protein-5/6 co-receptor (LRP5/6) [4]. This leads to the inhibition of glycogen synthase kinase-3β (GSK-3β) and proteasome-dependent degradation of β-catenin [5]. The stabilized β-catenin forms a complex with the T-cell specific transcription factor/lymphoid enhancer-binding factor (TCF/LEF) in the nucleus [6], and this β-catenin/TCF complex activates the expression of Wnt target genes, including *c-myc*, *cyclin D1*, and *MMP7* [7–9].

The Wnt signaling pathway is essential for maintaining cell homeostasis; however, aberrant Wnt signaling causes tumorigenesis and tumor progression [10]. In fact, various cancer cells reportedly exhibit mutations of Wnt signaling related-factors [11]. Mutations of adenomatous polyposis coli (APC) or β-catenin are found in approximately 90% of colorectal cancers [12]. Animal experiments have suggested that a mutation of APC or β-catenin induces tumorigenesis in mice [13, 14]. Moreover, patients with familial adenomatous polyposis (FAP) are reported to show loss-of-function mutations in the *APC* gene [15]. Thus, understanding the mechanism of the Wnt signaling pathway and the biological role of Wnt target genes are important with respect to developing efficacious cancer therapies and prophylaxis for these patients.

The stress response gene activating transcription factor 3 (ATF3) belongs to the ATF/CREB family of basic-leucine zipper transcription factors, and its expression is highly upregulated by a variety of factors causing stress, including ultraviolet radiation, genotoxic agents, and growth factors [16]. Indeed, ATF3 is induced as a target gene through various gene network pathways, such as JNK [17–19], p53 [20, 21], c-Myc [22, 23] and Smad [24]. It has been proposed that ATF3 is involved in several cellular adaptive responses [25].

Depending on the cellular context or cancer cell type, ATF3 reportedly exhibits a dichotomous role in determining cancer cell fate. For example, ATF3 plays an oncogenic role in malignant cell growth in Hodgkin’s lymphoma, and in prostate, skin and breast cancer cells [21, 26, 27]. In contrast, ATF3 acts as a tumor suppressor during the apoptotic cell death of prostate cancers and the metastasis of bladder cancer cells [28, 29]. This dichotomy in ATF3 functioning represents the diversity of the ATF3 activation pathways evoked by various signals.

In human colorectal cancer, ATF3 expression is reportedly lower than that in the surrounding non-cancerous tissue [30], and the RNAi-mediated silencing of ATF3 increased cancer cell migration both *in vitro* and *in vivo* [31]. Recently, we reported that ATF3 expression was induced by camptothecin through the p53 activation [32] and by zerumbone through the ATF4/CHOP pathway [33] and that this induction was correlated with the apoptotic death of human colorectal cancer cells [32, 33]. Hence, ATF3 might mediate tumor suppression through various signaling pathways in human colorectal cancer.

Cancer cell invasion is a trigger and key event for metastasis. Matrix metalloproteinases (MMPs) play a prominent role in the remodeling of the extracellular matrix by degrading its components. The elevated levels of expression of MMP-1, -2, -7, -9, and -13 correlate with adverse human colon cancer outcomes, while MMP-12 appears to have a protective function [34]. The activity of MMPs is regulated at multiple levels, including during transcription, mRNA half-life, secretion, localization, and inhibition of activity. Among these, the transcription of *MMP* genes is controlled in response to various stimuli, and many inducible *MMP* genes have AP-1 sites at both proximal and distal promoter regions, which could be recognized by Fos, Jun, and possibly ATF3 [35, 36]. Tissue inhibitors of metalloproteinases (TIMPs) comprise a family of four homologous protease inhibitors (TIMP 1–4), and contain AP-1 sites at their gene promoters [37, 38]. TIMPs bind MMPs in a stoichiometric 1:1 ratio and block the access of substrates to the catalytic domain of endopeptidases. The imbalance in the MMP/TIMP ratio has been shown to affect invasion and metastasis in some cancers, and is considered as a marker of cancer cell invasion [34, 37, 39].

Notably, ATF3 was recently reported to have been induced by the Wnt/β-catenin pathway and to play a role in the NDRG1-mediated suppression of metastasis of human breast and prostate cancer cells [40]. Furthermore, the transgenic overexpression of ATF3 in the mammary epithelium activates the canonical Wnt/β-catenin pathway and mammary tumor formation [41]. However, our knowledge of Wnt-dependent ATF3 gene expression and its biological role in human colorectal cancer is unknown.

In this study, we sought to understand the molecular mechanism and biological role of Wnt/β-catenin dependent ATF3 gene expression better by using HCT116 human cancer cells that were genetically engineered to possess a wild-type or mutant allele of the β-catenin gene loci [42, 43]. Our present study clearly showed that the stress response gene *ATF3* is a direct target for TCF4/β-catenin binding and plays a role in the suppression of cancer cell invasion and migration in human colorectal cancer cells.

## Materials and methods

### Plasmids, antibodies, and reagents

Plasmids for expressing or silencing ATF3 were constructed as described previously [32, 33], and expression plasmids encoding ΔNTCF4 and LEF1 DNA binding domain/β-catenin activation domain (LEF1ΔN-βCTA) were provided by Dr. Hecht at the Institute of Molecular Medicine and Cell Research, University of Freiburg, Germany [44]. Recombinant human Wnt3a was purchased from R&D systems (Minneapolis, MN, USA). The antibodies used included: anti-ATF3 (HPA001562), anti-Flag (F3165), and anti-β-actin (AC-74), which were purchased from Sigma-Aldrich (St. Louis, MO, USA). Anti-c-Myc (sc-764) and anti-cyclinD1 (sc-20044) were obtained from Santa Cruz Biotechnology (Santa Cruz, CA, USA), anti-β-catenin (610154) and anti-E-cadherin (610181) were obtained from BD Transduction Laboratories (Franklin Lakes, NJ, USA), anti-TBP (ab818) was obtained from Abcam (Cambridge, MA), and anti-TCF4 (6H5-3) was obtained from Millipore (Bedford, MA).

### Cell culture and media

Parental HCT116 and β-catWT or β-catMut cells that were hemi-targeted by homologous recombination were supplied by Dr. Vogelstein and Dr. Sekine [42, 43]. Human colon cancer cell lines LoVo, HT29, Caco-2, and SW480 were purchased from ATCC. HCT116 and HT29 cells were cultured in McCoy medium supplemented with 10% FBS and 1% penicillin/streptomycin, while HEK293T, SW480, and LoVo cells were cultured in DMEM medium supplemented with 10% FBS and 1% penicillin/streptomycin.

### Whole cell extracts and western blot analysis

Whole cell extracts were prepared for analysis by Western blotting using β-actin as a loading control, as described in a previous study [32], and images were captured by Las 500 (GE Healthcare) after using Luminol reagent (sc-2048, Santa Cruz Biotechnology) or ECL (GE Healthcare, Piscataway, NJ, USA).

### Quantitative reverse transcription (qRT) PCR

The total RNA was extracted using the RNeasy mini kit (Qiagen), and qRT-PCR was performed as described previously [45]. In this study, the following primer pairs were used: human *ATF3*, 5′-CTCCTGGGTCACTG- GTGTT-3′ (forward) and 5′-TCTGAGCCTTCAGTTCAGCA-3′ (reverse); human glyceraldehyde 3-phosphate dehydrogenase (*GAPDH*), 5′-GAGTCA- ACGGATTTGGTCGT-3′ (forward) and 5′-TTGATTTTGGAGGGATCTCG-3′ (reverse); human *Axin2*, 5′-TGGTGCCCTACCATTGACACA-3′ (forward) and 5′-GCAACATGGTCAACCCTCAAGA-3′ (reverse); human *DKK1*, 5′-TCCCC- TGTGATTGCAGTAAA-3′ (forward) and 5′-TCCAAGAGATCCTTGCGTTC-3′ (reverse); human *VEGFA*, 5′-GACCCTGGTGGACATCTTC-3′ (forward) and 5′-TGCATTCACATTTGTTGTGC-3′ (reverse); human *MMP7*, 5′-AGATGTG- GAGTGCCAGATGT-3′ (forward) and 5′-TAGACTGCTACCATCCGTCC-3′ (reverse); human *Id2*, 5′-TCAGCCTGCATCACCAGAGA-3′ (forward) and 5′-CTGCAAGGACAGGATGCTGAT-3′ (reverse); human *PPARD*, 5′-CCAGT- GGTTGCAGATTACAAGTATG-3′ (forward) and 5′-TTGTAGAGCTGAGTCT- TCTCAGAATAATAAG-3′ (reverse); human *c-jun*, 5′-GATACTAGCTATC- TAGGTGG-3′ (forward) and 5′-CATGCCACTTGATACAATCC-3′ (reverse); human *c-myc*, 5′-TACCCTCTCAACGACAGCAG-3′ (forward) and 5′-ACTCT- GACCTTTTGCCAGGA-3′ (reverse); and human *CCDN1*, 5′-CATCTACA- CCGACAACTCCATC-3′ (forward) and 5′-TCTGGCATTTTGGAGAGGAAG-3′ (reverse).

### Silencing or overexpression of ATF3 in HCT116 hemi-targeted cells

The retroviruses for siATF3-363 (5′-TGGAAAGTGTGTGAATGCTGAACT-3′), siATF3-493 (5′-AAGATGAGAGAAACCTCTTTA-3′), and siATF3-500 (5′-G- AAACCTCTTTATCCAACAGATA-3′) or ATF3 expression were prepared using pMX-puroII-U6 or pMX-neo-U6 vectors in Plat E cells [46]. HCT116 β-catWT or β-catMut cells were first transfected with pcDNA-mCAT encoding the ecotropic retrovirus receptor, and were infected by a mixture of the above retroviruses or by ATF3 expression, and selected by culturing in 10–40 μg/mL puromycin or 1000–5000 μg/mL neomycin (Invitrogen).

### Luciferase assay

The TOPflash reporter plasmid containing three copies of the TCF/LEF binding sites, and the FOPflash plasmid containing mutated TCF/LEF binding sites, were gifts from Dr. Sekine [43]. Various lengths of the 5’′-upstream region of the human *ATF3* reporter plasmid were used for the assay, as described previously [17]. The pATF3Luc-84 reporter plasmids containing internal deletions or point mutations were gifted by Dr. Seung Joon Baek [47]. The pATF3Luc-84 reporter plasmids with point mutations at the expected *TCF4* sequence (TATAAAAGGG), which were present at the -34 to -25 (mTCF4-1; 5′-TATAAAACCC-3′, mTCF4-2; 5′-TGCAAAAGGG-3′, mTCF4-3; 5′-TATAATAGGG-3′) sites, were prepared by subcloning each synthesized DNA. Each reporter plasmid was transfected into cells by SuperFect Transfection Reagent (Qiagen) and luciferase activity was measured using a Dual-Luciferase reporter assay system (Promega).

### Chromatin immunoprecipitation (ChIP) assay

ChIP assays were performed using a previously described protocol [48]. Briefly, cells were crosslinked by treatment with 0.5% formaldehyde for 20 min. After sonication, immunoprecipitation was performed at 4°C overnight using β-catenin antibody (sc-7199) or control mouse IgG (sc-2025). Pulled-down chromatins were reverse-crosslinked at 65°C overnight and purified by the QIAquick purification column (Qiagen). The DNA from the ChIP assay was measured by quantitative PCR (qPCR) using the following primer pairs: *ATF3 TBE*, 5′-CCCTTCCTCCGCTCCGTTCGG-3′ (forward) and 5′-CCTGGCTGCGTGCGACTGTGGC-3′ (reverse); *ATF3 P1-5K*, 5′-TGG- ACACACACACGGAAACT-3′ (forward) and 5′-GTCACATCTTCCCATC- TGATC-3′ (reverse); *GAPDH*, 5′-GCCCGATTTCTCCTCCG-3′ (forward) and 5′-GGACCTCCATAAACCCACTT-3′ (reverse); *Axin2 TBE*, 5′-AAGGCCCT- GCTGTAAAAGGT-3′ (forward) and 5′-GGGGGCTTTCTTTGAAGC-3′ (reverse); and *c-myc TBE*, 5′-GTGAATACACGTTTGCGGGTTAC-3′ (forward) and 5′-AGAGACCCTTGTGAAAAAAACCG-3′ (reverse). Fluorescence intensities were calculated using the formula: Fluorescence intensity = (IP average–IgG average)/(Input average).

### DNA affinity precipitation (DNAP) assay

Nuclear extracts (100 μg) from HCT116 cells were mixed with a biotinylated TCF4 site probe (100 pmol) in a buffer (HEPES-KOH pH 7.9, 80 mM KCl, 1 mM MgCl_2_, 0.2 mM EDTA, 0.5 mM DTT, 10%(W/V) glycerol, 0.1% Triton X-100) containing 1 μg poly(dI-dC) on ice for 30 min. Streptavidin agarose beads (Sigma-Aldrich) were added and the contents of the reaction mixture were mixed gently for 1 h. Beads were collected and washed three times by buffer, and the bound proteins were analyzed by Western blotting. The following sequences of the biotinylated TCF4 site were used: TCF4WT, 5′-TGAGGGCTATAAAAGGGGTGATGCA-3′; mTCF4-1, 5′-TGAGGGCTATA- AAACCCGTGATGCA-3′; mTCF4-2, 5′-TGAGGGCTGCAAAAGGGGTGATG- CA-3′, mTCF4-3, 5′-TGAGGGCTATAATAGGGGTGATGCA-3′.

### Wound healing assay

Cells were grown to confluency in 6-well plates and scratches were made on the plates using a sterile 200-μl pipette tip. Next, the plates were carefully washed with PBS, and fresh medium was added to them. They were incubated at 37°C, in 5% CO2 overnight. The gaps between scratches were photographed by a light microscope (Nikon) and measured with ImageJ software (NIH Image software program).

### Cell migration and invasion assays

Cell migration and invasion were assayed using 8 μm of the control and Matrigel filter pore inserts (Becton Dickinson Bioscience). Briefly, 2.5 × 10^5^ cells were suspended in McCoy medium without FBS and seeded into inserts on the plate, and subjected to transwell migration (without Matrigel) and invasion (with Matrigel) assays. These cells were incubated overnight at 37°C in 5% CO2. After incubation, migratory or invasive cells were stained using Diff-Quik (Sysmex) and washed with PBS. Five random fields per insert were photographed by using a light microscope.

### Cell growth in vitro

Cells (1 × 10^5^ cells) were seeded onto 60-mm dishes, and cells were counted by using a Muse Cell Analyzer (Merck Millipore) at day two, four, six, eight and ten.

### Xenograft assay

Six-week-old male athymic nude mice were purchased from Charles River (Charles River Laboratories International, Inc, MA, USA) and maintained under pathogen-free conditions for 1 week. Briefly, 1 × 10^7^ cells were prepared in 0.1 mL PBS and injected into the flank of the mice. After 4 weeks, the tumor was extirpated from the mice and immobilized by 10% formaldehyde, and the weight of each tumor was measured. All animal experiments were approved and conducted according the guidelines of the Committees of Animal Experiments (License Number 0150227A) and Recombinant DNA Experiments (License Number 2010-205C) of the Tokyo Medical and Dental University.

### qPCR of *MMP* and *TIMP* genes

Total RNA was extracted using the RNeasy mini kit (Qiagen), and cDNA was synthesized using the RT^2^ First Strand kit (Qiagen). A quantitative PCR (qPCR) of the reverse-transcribed cDNA was performed for estimating tumor metastasis (PAHS-028Z) by determining the expression of the *MMP* and *TIMP* genes using primer sets included in the kit.

### Gelatin zymography

Gelatin zymography was performed according to the manual of the Cosmo Bio Co. After 1 × 10^6^ cells were cultured using serum-free medium for 24h, the conditioned medium was collected, concentrated 50-fold using a Nanosep 10K centrifugal device (Pall Corporation, Washington, USA) and subjected to the gel plate of the Gelatin Zymo Electrophoresis Kit. The gel was incubated overnight at 37°C in an enzyme reaction buffer and stained with a protein staining solution.

### Statistical analysis

Statistical significance was assessed by comparing the mean (±S.E.) values with the Student’s t-test for independent groups.

## Results

### Activation of the canonical Wnt signaling pathway induces the expression of ATF3 in human colon cancer cells

**Fig 1:**
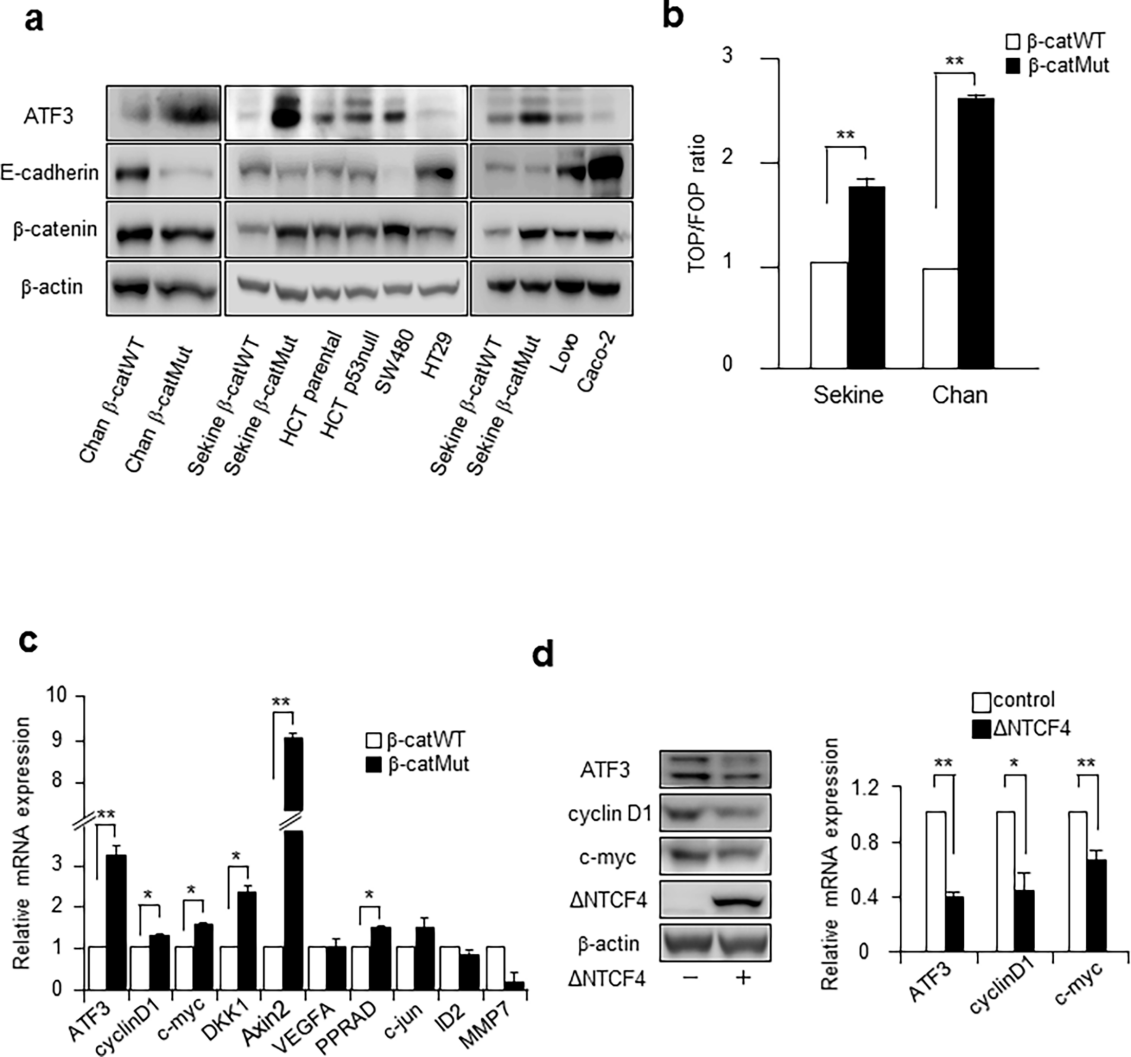
ATF3 is overexpressed in human colon cancer cells with aberrant Wnt/β-catenin signaling. (a) Several human colorectal cancer cells were assayed for detecting the ATF3, E-cadherin, and β-catenin proteins by Western blotting. HCT116 cells observed in the left (Chan β-catWT or β-catMut) or middle and right panels (Sekine's HCT116 β-catWT or β-catMut cells) were as in [42] or [43], respectively. Full-length blot images are shown in Fig a in S1 File. (b) TOPFOP reporter activity was measured in Chan’s and Sekine’s HCT116 β-catWT or β-catMut cells. Activity is represented as the TOP/FOP ratio relative to that of β-catWT/-, and data are represented as the mean ± S.E. values of three independent experiments. *, *p*<0.05 and **, *p*<0.01. (c) The levels of expression of mRNAs for ATF3 and putative Wnt targets in Chan’s HCT116 β-catWT or β-catMut cells were measured; they were normalized to those of GAPDH mRNA and were shown relative to the expression of β-catWT cells. Data shown are represented as the mean ± S.E. values of three independent experiments. *, *p*<0.05 and **, *p*<0.01. It is noted that the mRNA of *MMP7* gene in β-catMut cells could not to be measured by qRT-PCR because of unknown reasons. (d) Chan’s HCT116 β-catMut cells were transfected with the dominant negative TCF4 plasmid (ΔNTCF4), assayed for mRNAs, and analyzed by Western blotting. Full-length blot images are shown in Fig b in S1 File. ATF3, cyclin D1, and c-myc mRNA levels were measured, normalized to those of GAPDH mRNA, and shown relative to the expression of control β-catWT cells transfected with an empty vector. Data are represented as the mean ± S.E. values of three independent experiments. *, *p* < 0.05 and; **, *p* < 0.01.

To address whether the *ATF3* gene is regulated by the Wnt signaling pathway in human colon cancer cells, the ATF3 protein expression level was determined in several human colon cancer cell lines with APC or β-catenin mutations (S1 Fig). As shown in Fig 1A, HCT116 cells with mutant β-catenin (HCT116 β-catMut) showed a significantly elevated expression of ATF3 protein, compared to that in wild type cells (HCT116 β-catWT). These cells, Chan’s and Sekine’s cells, respectively, had been genetically engineered by homozygous recombination using different strategies in two laboratories [42, 43], to have either the β-catWT/- genotype that expressed only the WT allele or the β-catMut/- genotype that expressed only the mutant allele (S1C and S1D Fig). It should be noted that there were no visible differences in β-catenin levels (wt vs mutant) in Chan’s cells. This is consistent with the report by Chan *et al*. that showed that though the difference between the β-catenin levels in β-catWT and β-catMut cells was insignificant, mutant β-catenin showed increased binding to E-cadherin and greater nuclear localization [42]. The expression of ATF3 protein was also elevated in SW480 cells, in which the truncated *APC* gene lacked the region necessary for the degradation of β-catenin, leading to its accumulation [49, 50]. In contrast, ATF3 expression level was not elevated in HT29, LoVo, and Caco-2 cells, although the expression of β-catenin was increased, as shown in Fig 1A [49–51]. The reason why no correlation was observed between the expression of ATF3 and β-catenin in these cells is unclear, but it might be due to the increased expression of E-cadherin (Fig 1A) or the different genetic background(s) of each cell line, excluding their APC mutations. Therefore, in the following experiments, we focused on HCT116 β-catWT/- and β-catMut/- cells to characterize the β-catenin dependent transcription of ATF3 further, because only the β-catenin gene was genetically different between these cell lines. We next performed the TOPflash reporter assay in HCT116 β-catMut and β-catWT cells to determine their Wnt/β-cat signaling activity. Fig 1B shows that the mutant β-catenin allele is responsible for the aberrant activation of the Wnt pathway in these cells. Since Chan’s HCT116 cells showed more enhanced TOP/FOP activity than Sekine’s cells, we presented the data of experiments performed using Chan’s cells in the following studies. Data obtained using Sekine’ cells are also shown in supplementary figures. As seen in Fig 1C, the expression of mRNA of *ATF3* and other putative Wnt target genes was more elevated in Chan’s HCT116 β-catMut cells than that in β-catWT cells, suggesting that *ATF3* is a downstream target gene of Wnt signaling in these cells. Similar results were obtained for Sekine’s cells, as shown in S2A Fig. Wnt signaling involves two major pathways, canonical and non-canonical [2]. The canonical pathway induces the expression of target genes via the stabilized β-catenin/TCF complex. To examine whether the induction of *ATF3* involvesTCF4 via the Wnt pathway, a dominant negative TCF4 (ΔNTCF4) was transiently expressed in HCT116 β-catMut cells to inhibit its binding to the target sequence. As shown in Fig 1D, the mRNA and protein expression of ATF3, c-MYC, and cyclin D1 were repressed by ΔNTCF4, supporting the fact that *ATF3* induction is mediated by TCF4 as well as other Wnt target genes. Data obtained using Sekine’s cells are shown in S2B Fig.

### ATF3 expression is increased by Wnt3a and LiCl treatment in both HCT116 and HEK293T cells

**Fig 2:**
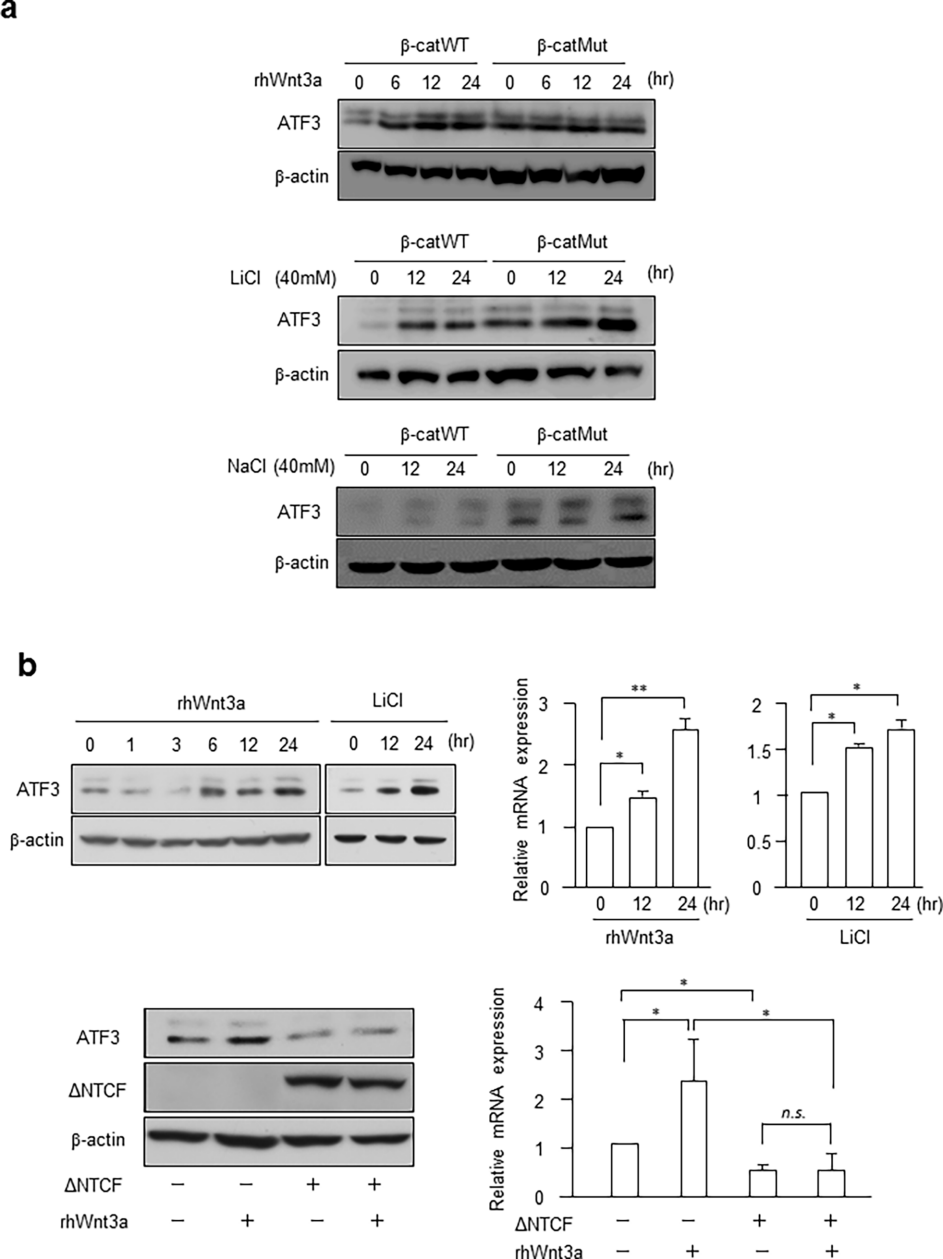
ATF3 expression is increased by Wnt and LiCl treatment in HCT116 and HEK293T cells. (a) Chan’s HCT116 β-catWT or β-catMut cells were treated with 100 ng/mL rhWnt3a, 40 mM LiCl or 40 mM NaCl and assayed for the ATF3 protein. Full-length blot images are shown in Fig c in S1 File. (b) HEK293T cells were treated as in (a) and assayed for ATF3 protein and mRNA. In the lower panel, cells were transfected with ΔNTCF4, treated by rhWnt3a, and assayed. Full-length blot images are shown in Fig d in S1 File. ATF3 mRNA levels were measured and normalized to those of GAPDH mRNA; the expression was shown relative to that of control cells without rhWnt3a or LiCl treatment (upper panel), or without rhWnt3a and ΔNTCF4 transfection (lower panel). Data are represented as the mean ± S.E. values of three independent experiments. *, *p* < 0.05; and **, *p* < 0.01.

To examine whether ATF3 is induced downstream of the Wnt signaling pathway, we treated Chan’s HCT116 β-catWT or β-catMut cells with recombinant Wnt3a or LiCl (Fig 2A). ATF3 protein was significantly induced in HCT116 β-catWT cells by these treatments. In contrast, in HCT116 β-catMut cells, the expression of ATF3 was induced in the absence of these stimulative treatments and did not increase further upon treatment with these stimuli. This suggested that in β-catMut cells, ATF3 expression is constitutively activated, because Wnt/β-catenin activation is aberrant in these cells due to the β-catenin mutation. Treatment of cells with 40 mM NaCl had no effect on ATF3 expression, supporting the fact that the effect of LiCl was observed because of the specific stimulation of the Wnt pathway. Similar results were obtained for Sekine’s cells, as shown in S2C Fig. Next, we examined the effect of these stimuli on ATF3 expression in normal HEK293T cells to address if ATF3 is regulated by the Wnt pathway in noncancerous cells. Fig 2B shows that Wnt3a and LiCl significantly induced the expression of ATF3 mRNA and protein. The transient expression of dominant negative TCF4 (ΔNTCF4) significantly repressed the induction of ATF3 by Wnt3a (Fig 2B lower panel). Therefore, these data strongly suggest that ATF3 is activated downstream of the Wnt pathway and its expression is regulated by β-catenin/TCF4 in HCT116 human colon cancer cells as well as normal HEK293T cells.

### β-catenin/TCF4 directly binds and activates the ATF3 gene promoter

**Fig 3:**
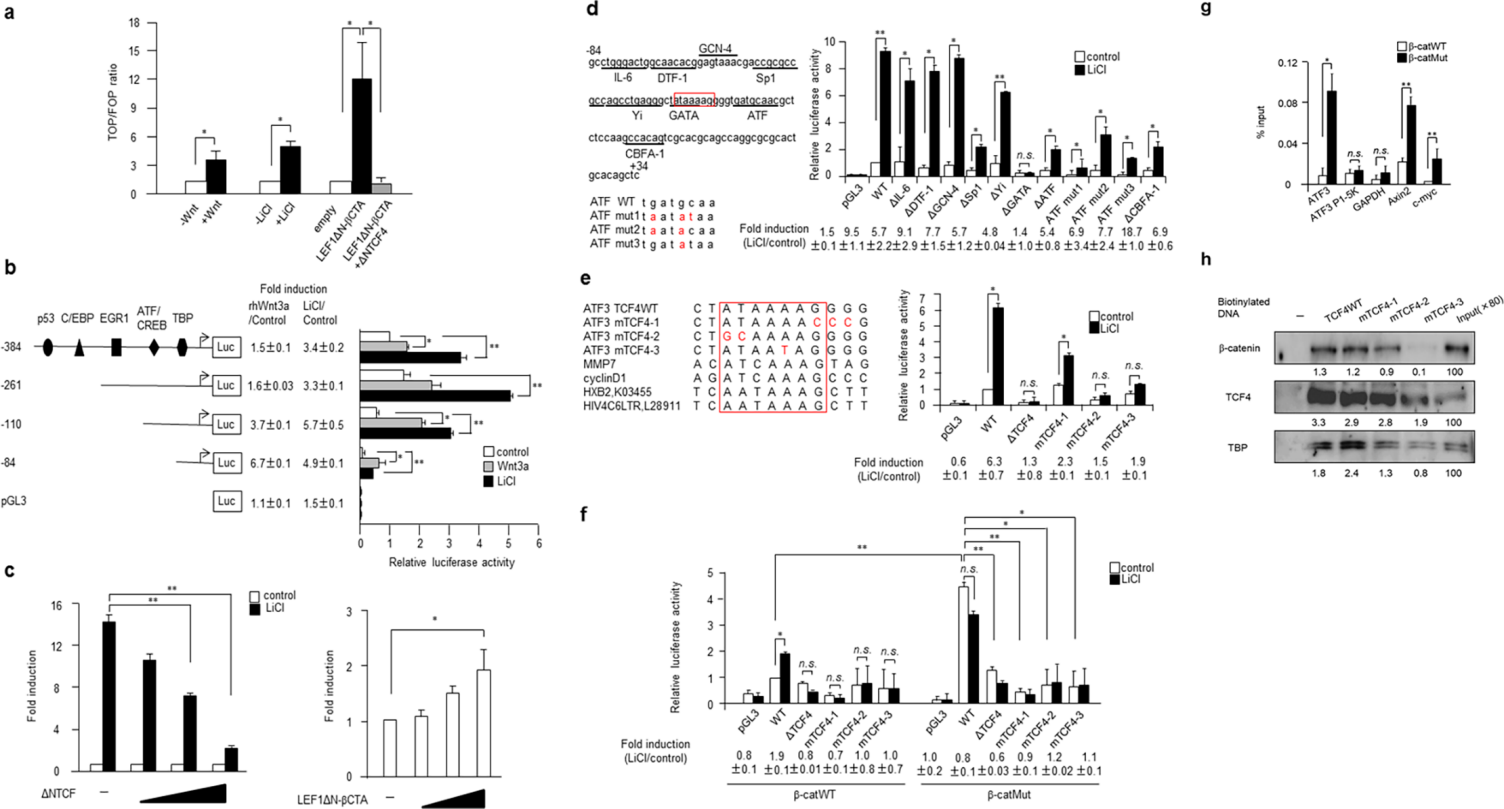
*ATF3* is a direct target for β-catenin/TCF4 binding onto the proximal gene promoter. (a) TOP/FOP reporter activity was assayed after the stimulation of HEK293T cells with rhWnt3a, LiCl, or co-expression of the β-catenin expression vector (LEF1ΔN-βCTA) with or without the dominant negative TCF4 plasmid (ΔNTCF4). Activity is shown as the TOP/FOP ratio relative to that of untreated or empty vector transfected cells. Data are represented as the mean ± S.E. values of three independent experiments. *, *p*<0.05. (b) HEK293T cells were transfected with pATF3Luc-384 or various 5′-deletion constructs of the human *ATF3* reporter plasmid (left panel), and assayed for luciferase activity with or without treatment by rhWnt3a (gray columns) or LiCl (black columns). The pATF3Luc-384 plasmid contains binding elements for p53, C/EBP, EGR1, ATF/CREB, and TBP. Fold induction is the ratio of the rhWnt3a- or LiCl-stimulated activity of each reporter to that of the pATF3Luc-384 reporter plasmid without treatment. Data are represented as the mean ± S.E. values of three independent experiments. *, *p*<0.05 and **, *p*<0.01. In (c), pATF3Luc-84 was assayed for reporter activity in HEK293T cells co-transfected with increasing amounts of ΔNTCF4 (left panel) or LEF1ΔN-βCTA (right panel). Fold induction is the activity relative to that without LiCl stimulation (left panel) or to that of transfection of an empty vector. Data are represented as the mean ± S.E. values of three independent experiments. *, *p*<0.05 and **, *p*<0.01. (d) The reporter activity of various deletion or point mutants of pATF3Luc-84 (left panel) was assayed in HEK293T cells before and after LiCl stimulation, and the activity relative to that of wild type pATF3Luc-84 (WT) in the absence of LiCl is shown in the right panel. Data are represented as the mean ± S.E. values of three independent experiments. *, *p*<0.05 and **, *p*<0.01. (e) The TCF-like sequence of the *ATF3* gene was aligned with those of putative genes for MMP7, cyclin D1, HXB2 and HIV4C6LTR of HIV LTR (left panel); its point mutations mTCF4-1, -2, and -3 were assayed for reporter activity in HEK293T cells before and after stimulation with LiCl (right panel). The activity relative to that of wild type pATF3Luc-84 (WT) in the absence of LiCl is shown. Data are represented as the mean ± S.E. values of three independent experiments. *, *p*<0.05 and **, *p*<0.01. In (f), various mutants of the TCF-like sequence of *ATF3* gene were also assayed in Chan’s HCT116 β-catWT or β-catMut cells, as shown in (e)**.** Data are represented as the mean ± S.E. values of three independent experiments. *, *p*<0.05 and **, *p*<0.01. (g) The β-catenin ChIP was performed in Chan’s HCT116 β-catWT (open columns) or β-catMut cells (black columns), and qPCR was performed to determine the presence of TBE (TCF4 binding motif) in the *ATF3*, *Axin2* and *c-myc* genes. *ATF3* P1-5K and *GAPDH* were the internal controls. Data are shown as the mean ± S.E. values of three independent experiments. *, *p*<0.05 and **, *p*<0.01. (h) Nuclear extracts of Chan’s HCT116 β-catMut cells were assayed using the DNAP assay as described in the Methods section, and the density of each protein was measured and its amount relative to that of the input is shown. Full-length blot images are shown in Fig e in S1 File.

The data above support the fact that *ATF3* is a transcriptional target gene of the Wnt canonical pathway. We first measured the TOPflash reporter activity of HEK293T cells (Fig 3A). It was activated by rhWnt3a, LiCl, or co-expression of the β-catenin expression vector, LEF1ΔN-βCTA, which was specifically inhibited by the dominant negative TCF4 plasmid (ΔNTCF4). To determine the important region(s) responsible for generating the Wnt response in the *ATF3* gene promoter, we next assayed the activity of the human *ATF3* reporter gene in HEK293 cells. As shown in Fig 3B, the region from -384 to +34 of the reporter gene pATF3Luc-384 and its 5′-deletion mutants [17] were activated by recombinant human Wnt3a and LiCl. While the activity of the plasmid pATF3Luc-84 was significantly lower than that of other plasmids, its fold induction was comparable with that of others, indicating that the Wnt3a or LiCl responsive element(s) is located more proximally to -84. As seen in Fig 3C, the activation of the pATF3Luc-84 reporter gene by LiCl was examined in HEK293T cells transfected with ΔNTCF4 or LEF1ΔN-βCTA. As shown in the left panel, the activation by LiCl was significantly suppressed by the co-expression of ΔNTCF4. In contrast, it was induced in a dose-dependent manner by the co-expression of LEF1ΔN-βCTA (Fig 3C, right panel). Thus, it was indicated that the sequence of the *ATF3* gene promoter from -84 to +34 is involved in transcriptional activation by Wnt signaling. As in the left panel of Fig 3D, this region contains various putative binding sites for IL-6, DTF-1, GCN-4, Sp1, Yi, GATA, ATF, and CBFA-1 [47]. We thus examined the activity of reporters containing deletion or point mutations in each of these binding sites in HEK293T cells. Fig 3D, right panel, shows that the deletion of merely the GATA site abolished activation by LiCl, indicating the critical role of this site in the Wnt response. On observing the sequence similarity of this region with that of other putative TCF motifs of *MMP7*, *cyclin D1* and *HIV LTR* such as HXB2 and HIV4C6LTR (left panel of Fig 3E) [8, 9, 52], we noticed a TCF4-like sequence in this region of the *ATF3* gene promoter, which was the same site reported in breast and prostate cells [40]. Thus, we constructed three mutations of these sites next, namely (Fig 3E, left panel), mTCF4-1, as in [40], and mTCF4-2 and -3, and measured their reporter activity after the stimulation of HEK293T cells with LiCl. The activation of these mutations by LiCl was significantly suppressed, whereas the wild-type reporter exhibited strong activation by LiCl that was significantly suppressed by ΔNTCF4 (Fig 3E, right panel). Next, we examined the activity of these mutations in Chan’s HCT116 β-catWT or β-catMut cells (Fig 3F). Wild type pATF3Luc-84 exhibited significant activation by LiCl in HCT116 β-catWT cells, whereas the activation achieved by LiCl was not as significant in TCF4 mutants. Notably, β-catMut cells in which the Wnt/β-catenin pathway was constitutively activated, showed a higher reporter activity in the absence of stimulation, and were not activated further by LiCl. Similar results were observed in Sekine’s cells, as shown in S3A Fig. To further address whether β-catenin directly binds to the *ATF3* gene promoter, the chromatin immunoprecipitation (ChIP) assay using the anti-β-catenin antibody was performed in Chan’s HCT116 β-catWT and β-catMut cells. Fig 3G shows that β-catenin was specifically recruited to this region of the *ATF3* gene, but not to the -5K region of the *ATF3* gene and the *GAPDH* gene used as the negative control [48]. As a positive control, β-catenin was specifically recruited to the Wnt target genes *c-myc* and *Axin2* [53, 54]. Notably, more β-catenin was recruited to these TCF4 motifs in Chan’s β-catMut cells than that in β-catWT cells, in which increased expression of E-cadherin might have prevented the nuclear localization of β-catenin (Fig 1A and [42]). Data for Sekine’s cells are shown in S3B Fig. Next, the specific binding of β-catenin and TCF4 proteins to the TCF4-like sequence of *ATF3* gene was also examined by a DNA affinity precipitation (DNAP) assay. As shown in Fig 3H, these proteins bound to wild type TCF4 sequence, and the binding was decreased to varying degrees with mutant TCF4, while binding to mTCF4-3 was remarkably decreased. The TATA-binding protein (TBP) was also observed to bind to this proximal sequence of the gene promoter. Results for Sekine’s cells are shown in S3C Fig. Taken together, these data strongly support the fact that the β-catenin/TCF4 complex is recruited to the TCF4 element of the proximal human *ATF3* gene promoter and mediates *ATF3* gene expression, which is consistent with the results of a previous study conducted using human prostate and breast cancer cells [40].

### Wnt-dependent ATF3 expression did not affect HCT116 cancer cell proliferation but inhibited the migration and invasion

**Fig 4:**
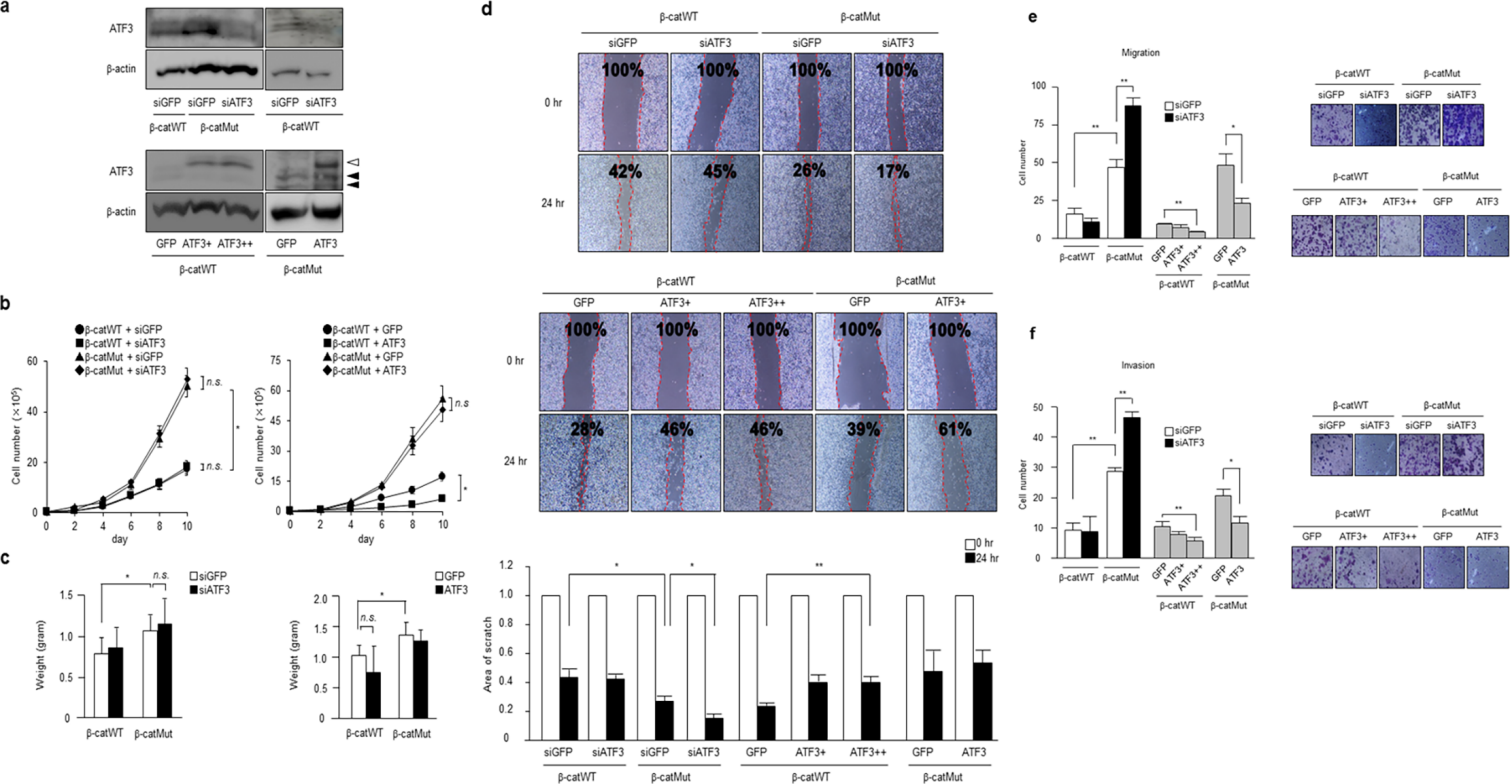
ATF3 represses cell migration and invasion of HCT116 cells. (a) ATF3 was knocked down (upper panel) or overexpressed (lower panel) in Chan’s HCT116 β-catMut or β-cat WT cells, as described in the Methods section, and cell extracts of the established cell clones were analyzed for ATF3 protein by Western blotting. ATF3+ and ++ represent the amounts of retrovirus used for overexpressing ATF3. The concentration of retroviruses used for (++) was two times more than that for (+). Open and black arrowheads indicate the bands of transfected Flag-tagged ATF3 and endogenous ATF3, respectively. Full-length blot images are shown in Fig f in S1 File. (b) The growth of cells, as established above, was measured by the *in vitro* proliferation assay, and cells were counted on the days indicated. Data are represented as the mean ± S.E. values of three independent experiments. *, *p*<0.05 and **, *p*<0.01. (c) As established above, cells were subjected to *in vivo* xenograft assay using the protocol described in the Methods section. In this assay, the weight of tumors in nude mice was measured 4 weeks after injection. Data are represented as the mean ± S.E. values of three independent experiments. *, *p*<0.05 and **, *p*<0.01. In (d), Chan’s HCT116 β-catMut or β-cat WT cells were assayed for determining the extent of wound healing and the scratch area was measured. The scratch area is shown as the relative ratio to that at time zero. In the lower panel, data are represented as the mean ± S.E. values of three independent experiments. *, *p*<0.05 and **, *p*<0.01. The cell migration (e) or invasion (f) assay was performed, as described in the Methods section. Typical photographs of cell staining are shown (right panel), and represent migrated or invaded cells, respectively. Results are also quantified using ImageJ software and the cell numbers represent the mean ± S.E. values of three independent experiments. *, *p* < 0.05 and; **, *p* < 0.01.

Previous studies have reported that the putative Wnt target genes play oncogenic roles in cancer cell proliferation, migration, and invasion [10], whereas some genes such as *DKK1* function as tumor suppressors. To elucidate the biological role of Wnt-dependent ATF3 expression in human colon cancer cells further, *ATF3* expression was knocked down or overexpressed in Chan’s HCT116 β-catMut or β-catWT cells (Fig 4A). As seen in Fig 4B, *in vitro* proliferation of β-catMut cells was significantly accelerated compared to that of β-catWT cells, which was consistent with Wnt activation in β-catMut cells [42]. However, knockdown of ATF3 in β-catMut or β-catWT cells had no apparent effects on *in vitro* cell proliferation (Fig 4B, left panel). Next, we examined the effect of ATF3 overexpression. In the right panel of Fig 4B, it had no significant effect on β-catMut cells, whereas a small but significant inhibition of the in-vitro proliferation of β-catWT cells was observed. Next, we assayed these cells for *in vivo* xenograft activity in nude mice. In Fig 4C, a significant increase in tumor size was observed for β-catMut cells compared to that for β-catWT cells. However, neither the knockdown nor overexpression of ATF3 in these cells had a significant effect on tumor growth. Thus, contrary to the report by Hackle *et al*. [31], ATF3 had no significant effect on i*n vitro* and *in vivo* proliferation of HCT116 β-catMut or β-catWT cells. Results with Sekine’s cells are shown in S4A–S4C Fig. Next, cell migration activity was examined using a wound healing assay. As seen in Fig 4D, the scratch area was significantly reduced for Chan’s HCT116 β-catMut cells compared to that for β-catWT cells, which is consistent with the higher migration activity in β-catMut cells [42]. *ATF3* knockdown in β-catMut cells significantly reduced the scratch area, while overexpression of ATF3 in β-catWT cells significantly increased the scratch area (Fig 4D, lower panel), indicating that ATF3 mediates the suppression of cell migration. Cell migration and invasion were further examined using control and Matrigel filter pore inserts, respectively. As shown in Fig 4E and 4F, Chan’s β-catMut cells exhibited higher migration and cell invasion activities, and *ATF3* knockdown significantly increased their migration and invasion, whereas ATF3 overexpression inhibited migration and invasion of both β-catWT and β-catMut cells. Data for Sekine’s cells are shown in S4E and S4F Fig. These data, altogether, indicate that though Wnt-dependent ATF3 expression did not affect cell proliferation; it had a tumor suppressive role because it negatively affected the migration and invasion of HCT116 colon cancer cells.

### ATF3 regulates the mRNA expression of some *MMP* and *TIMP* genes and their ratio, but not MMP2 and 9 protein levels in HCT116 cells

**Fig 5:**
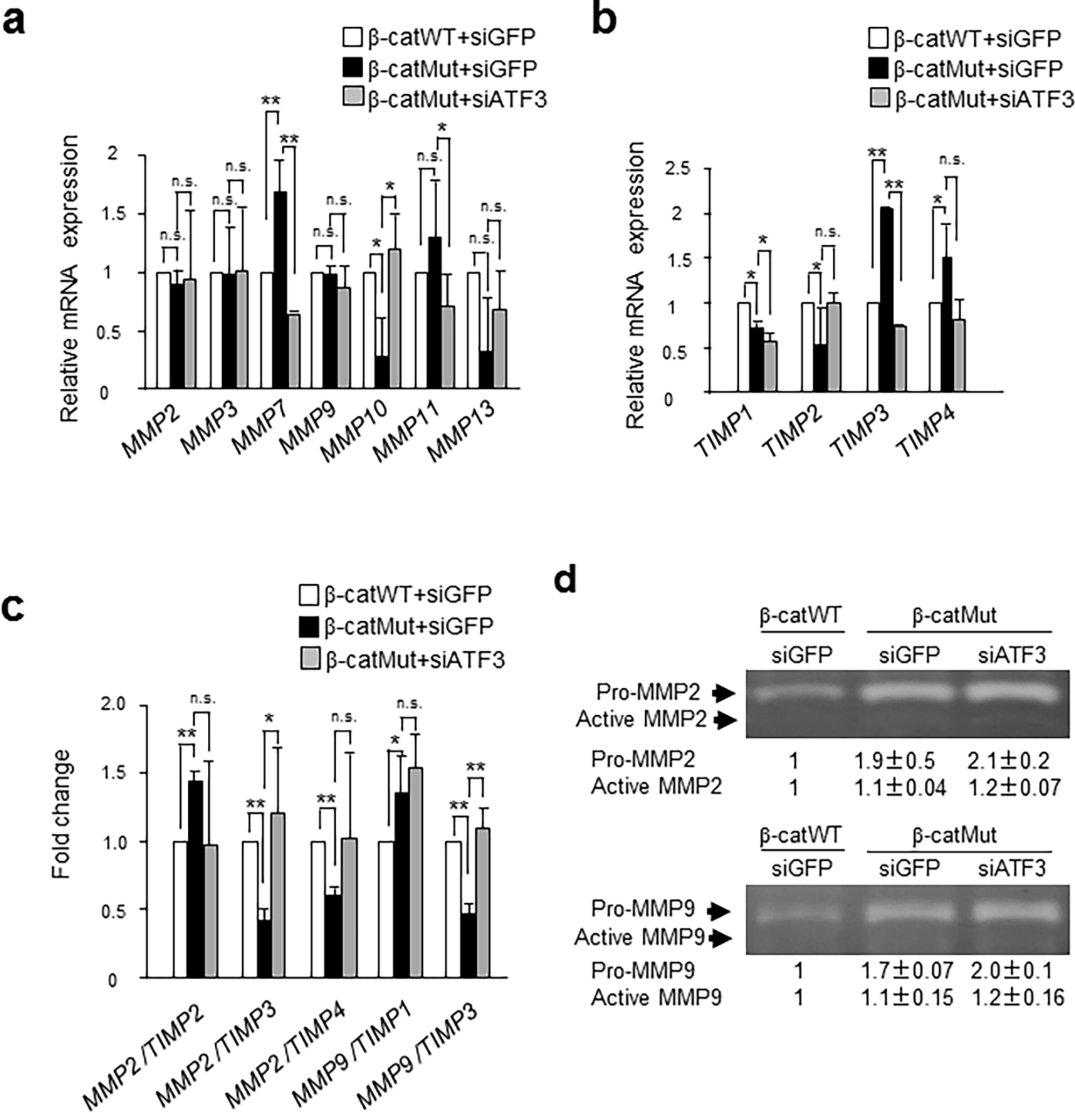
ATF3 regulates *MMPs*, *TIMPs* and *MMP/TIMP* mRNA expression but not MMP2 and MMP9 protein levels in HCT116 cells. The mRNA expression level was measured, normalized to that of GAPDH mRNA, and compared with those of HCT116 β-catWT (open bar) and β-catMut (closed bar) or β-catMut cells exhibiting ATF3 silencing (gray bar) for *MMPs* (a), *TIMPs* (b), and *MMP*/*TIMP* ratio (c), respectively. Data are shown as relative expression (a, b) or fold change (c) compared to β-catWT cells. Data are represented as the mean ± S.E. values of three independent experiments. *, *p*<0.05 and **, *p*<0.01. In (d), the MMP2 (upper panel) and MMP9 proteins (lower panel) of the β-catWT and β-catMut cells, or β-catMut cells with silenced *ATF3* gene were stained for determining the pro and active forms by gelatin zymography, as described in the Methods section. The relative amount of each form to that of β-catWT cells was measured from the density of each band obtained by ImageJ software. Full-length gel images are shown in Fig g in S1 File. Data are represented as the mean ± S.E. values of three independent experiments.

*MMP* and *TIMP* genes have been shown to regulate cancer cell invasion, and ATF3 is reported to reduce the ability to migrate by regulating the *MMP* and *TIMP* expression in human glioblastoma cells [55]. To address if ATF3 regulates the expression of *MMPs* or *TIMPs* in HCT116 cells, we performed PCR array analysis of Chan’s β-catWT, β-catMut, and β-catMut cells after ATF3-knockdown. As shown in Fig 5A, the expression of *MMP2*, *3*, and *9* genes was not altered among these cells, but the expression of *MMP7* was significantly elevated in β-catMut cells and was down-regulated by ATF3 knockdown, suggesting that the *MMP7* gene is probably activated by ATF3. The activation of *MMP11* was also observed in β-catMut cells while the decrease in its expression after ATF3 knockdown was not statistically significant. The activation of *MMP7* in β-catWT cells was consistent with that in a previous report [34] and may be correlated to the higher invasive activity of these cells. In contrast, mRNAs of *MMP10 and 13* were significantly reduced in β-catMut cells and rescued by ATF3 knockdown, while the change in levels of *MMP13* mRNA was statistically insignificant, suggesting that the *MMP10* and *13* genes were suppressed by ATF3 in β-catMut cells. As shown in Fig 5B, the expression of *TIMP3* and *4* genes was significantly elevated in β-catMut cells and that of *TIMP3* was significantly reduced after ATF3 knockdown, while the decrease in *TIMP4* expression was statistically insignificant. This suggests that ATF3 activated *TIMP3* and *4* genes to inhibit MMP activity in β-catMut cells. *TIMP2* gene was significantly downregulated in β-catMut cells while its downregulation was not significantly improved after ATF3 knockdown. In human colon cancer, it has been reported that the expression of *MMP2*, *7*, and *9* correlates with an increased progression of cancer [34]. However, the expression of the *MMP2* and *9* genes was neither altered between β-catWT and β-catMut cells, nor affected by *ATF3* gene silencing (Fig 5A). Thus, we examined whether the ratio of mRNA expression of *MMP2* and *MMP9* to that of *TIMPs* was affected by ATF3. As shown in Fig 5C, the *MMP2*/*TIMP2* ratio was significantly elevated in β-catMut cells compared to that in β-catWT cells and was decreased by ATF3 knockdown. In contrast, the ratio of mRNA expression of *MMP2/TIMP3*, *MMP2*/*TIMP4*, and *MMP9*/*TIMP3* was significantly decreased in β-catMut cells, and this reduction was rescued by *ATF3* gene silencing, while the recovery of the *MMP2*/*TIMP4* ratio was insignificant. These data indicate that ATF3 downregulates the mRNA expression ratio of *MMP2/TIMP3*, *MMP9/TIMP3*, and possibly *MMP2/TIMP4* in HCT116 β-catMut cells.

ATF3 has been reported to affect the degradation of MMP2 at the protein level via the ATF3/MDM2/MMP-2 complex in esophageal cancer cells [56]. Thus, we addressed if ATF3 affects the MMP2 and 9 protein expression in HCT116 cells. Fig 5D showed that the expression of MMP2 and 9 proteins, in both proactive and active forms, was highly elevated in β-catMut cells than that in β-catWT cells, but their level was not altered or was only slightly enhanced by ATF3 knockdown, showing that ATF3 is unlikely to regulate *MMP2* and *9* gene mRNA expression and protein stability in HCT116 cells.

## Discussion

The Wnt/β-catenin signaling pathway plays important roles in carcinogenesis and tumor progression [1, 10], and most of the Wnt target genes function as oncogenes to accelerate cancer growth and invasion. Indeed, HCT116 human colon cancer cells with β-catenin mutations exhibit a higher activity with regard to cell proliferation, migration, and invasion (Fig 4). On the other hand, some of the Wnt targets have been shown to have adverse effects and counteract oncogenic Wnt/β-catenin activity [57, 58].

In this study, we clearly show that *ATF3* is a direct transcriptional target gene of the Wnt/β-catenin pathway in normal human and HCT116 colon cancer cells. This is consistent with the findings of a previous report by Liu *et al*., which showed that ATF3 is induced by Wnt or the ectopic expression of β-catenin and TCF in breast and prostate cancer cells, and identified the β-catenin/TCF binding consensus sequence at the proximal promoter of the *ATF3* gene [40].

In HCT116 human colorectal cancer cells, the expression of ATF3 mRNA and protein were correlated to β-catenin gene mutations (Fig 1). More strikingly, in both Chan’s and Sekine’s HCT116 β-catMut cells, ATF3 and some of the putative Wnt targets were expressed at a significantly higher level than the wild-type (Fig 1C) gene and their expression was specifically suppressed by dominant negative TCF4 (ΔNTCF4, Fig 1D). We identified a TCF4 binding element at the -34 to -28 region of the *ATF3* gene promoter, and this was supported by the results of the ChIP and DNA affinity precipitation (DNAP) assays (Fig 3). The TCF4 binding element (TBE) of the *ATF3* gene, ATAAAAG, was not completely homologous to the general motif A/TA/TCAAAG [59]. However, a recent study of the HIV *LTR* gene showed that its TBE motif is AATAAAG [52], which resembles that of the *ATF3* gene (Fig 3E). Moreover, the DNAP assay showed that β-catenin, TCF4, and TATA-binding protein (TBP) were strongly bound to the TBE region (Fig 3H). This supports the fact that TBE overlaps with the TATA box of the *ATF3* gene and that the β-catenin/TCF4 complex is localized in close proximity to TBP, because it has been reported that both TCF4 and TBP bind to the minor groove of DNA [52, 60]. While we could not describe how the β-catenin/TCF4 complex and TBP interact at this region of the *ATF3* gene promoter, it is likely that the β-catenin/TCF4 complex somehow accelerates the transcriptional initiation or elongation because of their proximity around the proximal gene promoter.

We have previously reported that ATF3 is a downstream target of c-myc in the serum response of rat fibroblasts and that its activation is mediated by the formation of the ATF2/c-Jun/c-Myc ternary complex at the ATF/CRE site of the proximal promoter region of *ATF3* [22]. This site is located at the -92 to -84 region of the ATF3 gene promoter, which is very close to the TCF4 binding element at the -34 to -28 region, as determined in this study (Fig 3). More recently, Mathiasen *et al*. showed that ATF3 is a target of ATF2 and c-Myc, and that it is required for ras transformation of JNK-deficient mouse embryonic fibroblasts [23]. These findings suggest that ATF3 plays a role in oncogenesis, possibly through the JNK/ERK pathway. Considering that c-Myc is a putative target gene of the Wnt/β-catenin pathway, we first postulated that ATF3 is induced via c-Myc. However, in this study, it was clearly shown that *ATF3* is a direct target of β-catenin/TCF4 binding, leading to the possibility that *ATF3* is activated by both MAPK/JNK and Wnt signals to mediate their biological functioning in colorectal cancer in a context-dependent manner [61, 62].

We studied the biological role of ATF3 in human colorectal cancer cells. Previous studies have shown that ATF3 expression is correlated with cancer cell death or suppressor of cell growth and metastasis in HCT116 cells [30, 31, 32, 33, 63]. In this study, however, ATF3 suppressed the *in vitro* migration and invasion of HCT116 β-catMut or β-cat WT cells (Fig 4D, 4E and 4F), whereas ATF3 gene silencing did not significantly affect *in vitro* cell proliferation and *in vivo* tumor growth in xenograft assays (Fig 4B and 4C). In contrast, Hackl *et al*. reported an increase in the tumor growth for HCT116 cells upon treatment with shATF3 [31]. We do not know why this experiment could not be reproduced in our study; however, it is of note that we employed genetically engineered HCT116 β-catMut cells, while Hackl *et al*. used parental β-catWT/Mut cells in their experiments.

DKK1 and sFRP are some of the negative regulators of the Wnt/β-catenin targets, which suppress cancer cell proliferation [57, 58]. This study proposed that in cancer cells with β-catenin mutations, such as HCT116 cells, *ATF3* transcription is constitutively activated through the β-catenin/TCF4 binding, but over-expressed ATF3 exhibits a tumor suppressive function by repressing cancer migration and invasion.

ATF3 regulated the genetic expression of *MMP* and *TIMP* mRNAs positively or negatively (Fig 5A and 5B). Though it is not known whether this effect was mediated directly by ATF3, many of the *MMP* and *TIMP* genes contain AP1 sites at their gene promoters [37, 38]; ATF3 functions as a transcriptional activator or repressor depending on hetero- or homodimers of ATF3, in different cellular contexts [16, 36, 64]. Therefore, it might be possible that the regulation of mRNA expression of *MMP* and *TIMP* and their ratios by ATF3 collectively reduced invasion and migration in β-catMut HCT116 cells, as seen in other cells [34, 65, 66]. A previous report showed that ATF3 degrades the MMP protein by a proteasome-dependent pathway in esophageal cancer [56]; however, ATF3 was unlikely to regulate the expression of *MMP2* and *9* genes or the MMP2 and 9 protein stability in HCT116 cells, in this study.

Finally, we demonstrated that ATF3 is a direct target gene of β-catenin/TCF4 binding and negatively regulates tumor progression in human colon cancer cells. Since the Wnt signaling pathway is one of those that are frequently mutated in human colorectal cancer [12, 67], ATF3 is a candidate biomarker and target for cancer treatment and prevention.

## Supporting information

S1 Fig *APC* or *β-catenin* genes in various human colon cancer cells. Diagrams of the *APC* and β-catenin genes of HCT116 human colorectal cancer cells (a), and the Caco-2, Lovo, HT29, and SW480 cell lines (b) are shown. The wild type *APC* gene contains three 15AARs and seven 20AARs for β-catenin binding (CID domain), and three SAMPs for Axin binding. The wild-type β-catenin gene has phosphorylation sites in GSK3β that are required for proteasome-dependent degradation. WT, Mut, and WT/Mut represent wild, mutant, and heterozygous types of each gene, respectively. The structures of the wild type and targeted alleles of the β-catenin gene in Chan’s and Sekine’s HCT116 cells are shown in (c) and (d), respectively. These cells have been genetically engineered by homozygous recombination using different strategies in two laboratories [42, 43], to have either the β-catWT/- genotype that expresses only the WT allele or the β-catMut/- genotype that expresses only the mutant allele.
(TIF)

S2 Fig ATF3 is induced by canonical Wnt signaling pathway in Sekine's HCT116 β-catMut cells. (a) The levels of expression of ATF3 and other Wnt target gene mRNAs were determined and normalized to those of GAPDH mRNA. (b) HCT116 β-catMut cells were transfected with dominant negative TCF4 plasmid (ΔNTCF4), and cell extracts were assayed for ATF3, cyclin D1, and c-myc proteins or mRNAs. Full-length blot images are shown in Fig a in S2 File. (c) Cells were treated with 100 ng/mL recombinant human Wnt3a (rhWnt3a), 40 mM LiCl, or 40 mM NaCl for the indicated time, and assayed for the ATF3 protein by Western blotting. Full-length blot images are shown in Fig b in S2 File. Data are represented as the mean ± S.E. values of three independent experiments. *, *p* < 0.05 and **, *p* < 0.01.
(TIF)

S3 Fig *ATF3* is a direct target of Wnt signaling and the TCF4/β-catenin complex is recruited onto the proximal *ATF3* gene promoter in Sekine's HCT116 β-catMut cells. (a) HCT β-cat Mut, Wt or parental cells were transfected with wild-type gene or each mutation of the TBE of pATF3Luc-84 and treated with 40 mM LiCl for 24 h, and its reporter activity was assayed. (b) β-catenin ChIP assay was performed in HCT116 β-catWt (open columns) or β-catMut (black columns) cells by using a primer set for the putative TBE region on ATF3 gene. ATF3 P1-5K, which is present 5 kb upstream of the ATF3 P1 gene promoter, and GAPDH primers are the negative controls. Axin2 and c-myc primers are positive controls. (c) Nuclear extracts of HCT116 β-cat Mut cells were mixed with each biotinylated DNA probe and assayed for β-catenin, TCF4, and TBP proteins by Western blotting. The density of the band was measured and its relative input is shown. Full-length blot images are shown in Fig c in S2 File. Data are represented as the mean ± S.E. of values three independent experiments. *, p < 0.05 and **, p < 0.01.
(TIF)

S4 Fig ATF3 represses cell migration and invasion in Sekine's HCT116 β-catMut cells. (a) ATF3 was knocked down or overexpressed in HCT116 β-catMut or β-catWT cells, respectively, and the growth of each cell *in vitro* (b) or *in vivo* (c) was measured as described in the Methods section. Open and black arrowheads indicate the bands of transfected Flag-tagged ATF3 and endogenous ATF3, respectively. Full-length blot images are shown in Fig d in S2 File. In the xenograft assay, the weight of tumors in nude mice was measured 4 weeks after injection (c). In (d), cells were assayed for wound healing and the scratch area was measured, as detailed in the Methods section. The cell migration (e) or invasion (f) assay was performed as described in the Methods section. All the data are represented as the mean ± S.E. values of three independent experiments. *, *p* < 0.05 and **, *p* < 0.01.
(TIF)

S1 File Full length Western blot and MMP assay gel images of Figs. (a) and (b) are blots of Fig 1A and 1D, respectively. (c) and (d) are blots of Fig 2A and 2B, respectively. (e) represents blots and DNAP assay of Fig 3H, and (f) are blots of Fig 4A. (g) MMP assay gel shown in Fig 5D.
(PDF)

S2 File Full length Western blot images of S Figs. (a) and (b) are blots of S2B and S2C Fig, respectively. (c) represents blots and DNAP assay of S3C Fig. (d) is blots of S4A Fig.
(PDF)
