# Supplementary figures and images for: The stress response gene ATF3 is a direct target of the Wnt/β-catenin pathway and inhibits the invasion and migration of HCT116 human colorectal cancer cells
Source: PLoS One. 2018 Jul 2;13(7):e0194160. doi: 10.1371/journal.pone.0194160 (PMC6028230; doi:10.1371/journal.pone.0194160)

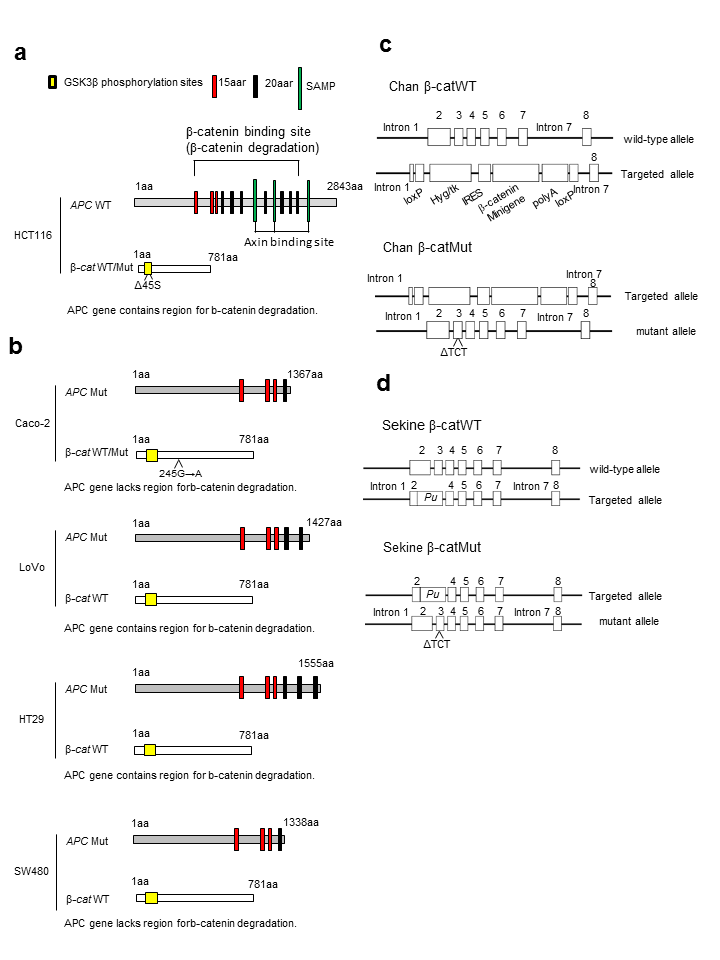

Supplement: S1 Fig — Diagrams of the APC and β-catenin genes of HCT116 human colorectal cancer cells (a), and the Caco-2, Lovo, HT29, and SW480 cell lines (b) are shown. The wild type APC gene contains three 15AARs and seven 20AARs for β-catenin binding (CID domain), and three SAMPs for Axin binding. The wild-type β-catenin gene has phosphorylation sites in GSK3β that are required for proteasome-dependent degradation. WT, Mut, and WT/Mut represent wild, mutant, and heterozygous types of each gene, respectively. The structures of the wild type and targeted alleles of the β-catenin gene in Chan’s and Sekine’s HCT116 cells are shown in (c) and (d), respectively. These cells have been genetically engineered by homozygous recombination using different strategies in two laboratories [42, 43], to have either the β-catWT/- genotype that expresses only the WT allele or the β-catMut/- genotype that expresses only the mutant allele. (TIF) [file pone.0194160.s001.TIF]

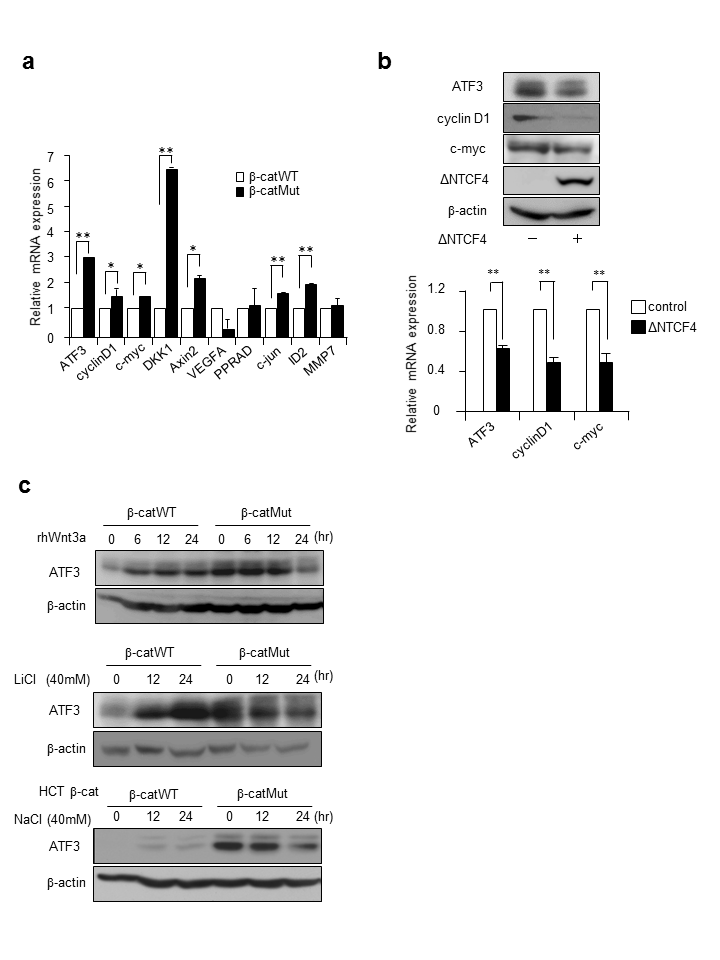

Supplement: S2 Fig — (a) The levels of expression of ATF3 and other Wnt target gene mRNAs were determined and normalized to those of GAPDH mRNA. (b) HCT116 β-catMut cells were transfected with dominant negative TCF4 plasmid (ΔNTCF4), and cell extracts were assayed for ATF3, cyclin D1, and c-myc proteins or mRNAs. Full-length blot images are shown in Fig a in S2 File. (c) Cells were treated with 100 ng/mL recombinant human Wnt3a (rhWnt3a), 40 mM LiCl, or 40 mM NaCl for the indicated time, and assayed for the ATF3 protein by Western blotting. Full-length blot images are shown in Fig b in S2 File. Data are represented as the mean ± S.E. values of three independent experiments. *, p < 0.05 and **, p < 0.01. (TIF) [file pone.0194160.s002.TIF]

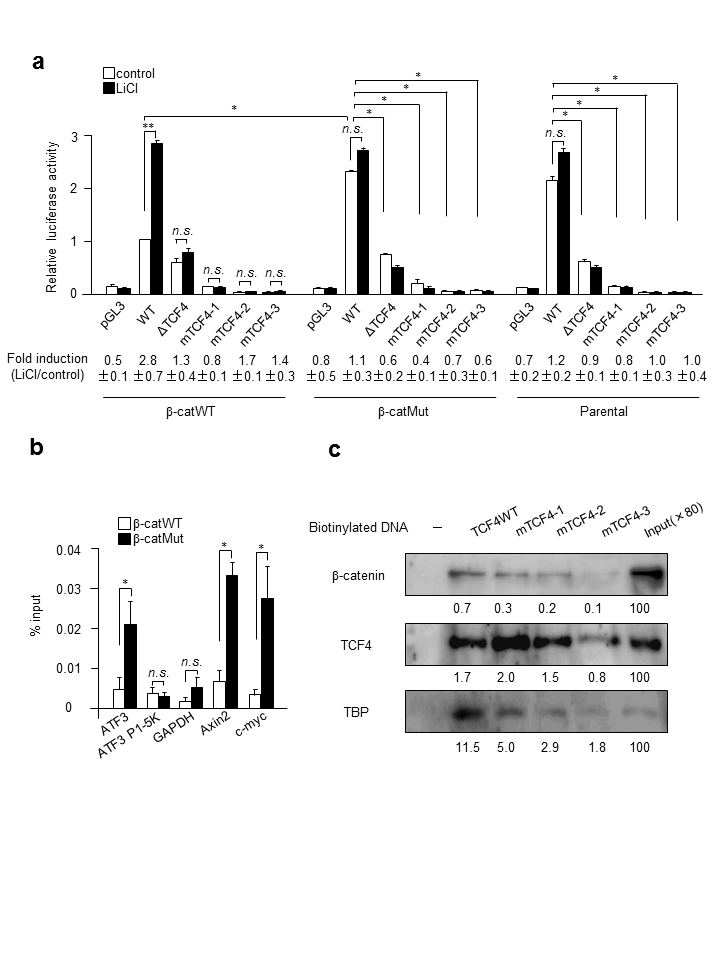

Supplement: S3 Fig — (a) HCT β-cat Mut, Wt or parental cells were transfected with wild-type gene or each mutation of the TBE of pATF3Luc-84 and treated with 40 mM LiCl for 24 h, and its reporter activity was assayed. (b) β-catenin ChIP assay was performed in HCT116 β-catWt (open columns) or β-catMut (black columns) cells by using a primer set for the putative TBE region on ATF3 gene. ATF3 P1-5K, which is present 5 kb upstream of the ATF3 P1 gene promoter, and GAPDH primers are the negative controls. Axin2 and c-myc primers are positive controls. (c) Nuclear extracts of HCT116 β-cat Mut cells were mixed with each biotinylated DNA probe and assayed for β-catenin, TCF4, and TBP proteins by Western blotting. The density of the band was measured and its relative input is shown. Full-length blot images are shown in Fig c in S2 File. Data are represented as the mean ± S.E. of values three independent experiments. *, p < 0.05 and **, p < 0.01. (TIF) [file pone.0194160.s003.TIF]

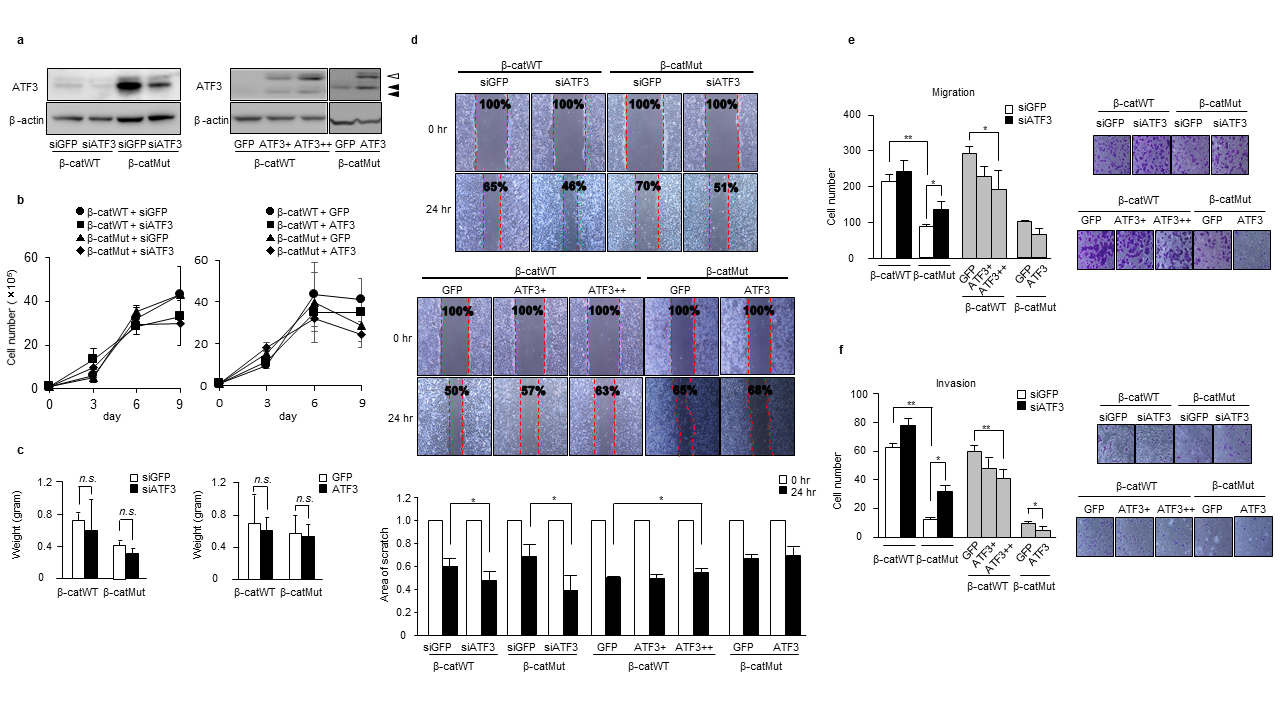

Supplement: S4 Fig — (a) ATF3 was knocked down or overexpressed in HCT116 β-catMut or β-catWT cells, respectively, and the growth of each cell in vitro (b) or in vivo (c) was measured as described in the Methods section. Open and black arrowheads indicate the bands of transfected Flag-tagged ATF3 and endogenous ATF3, respectively. Full-length blot images are shown in Fig d in S2 File. In the xenograft assay, the weight of tumors in nude mice was measured 4 weeks after injection (c). In (d), cells were assayed for wound healing and the scratch area was measured, as detailed in the Methods section. The cell migration (e) or invasion (f) assay was performed as described in the Methods section. All the data are represented as the mean ± S.E. values of three independent experiments. *, p < 0.05 and **, p < 0.01. (TIF) [file pone.0194160.s004.tif]

(a)

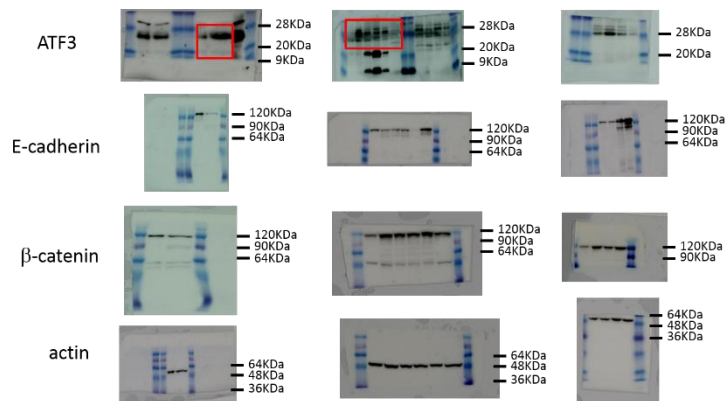

(b)

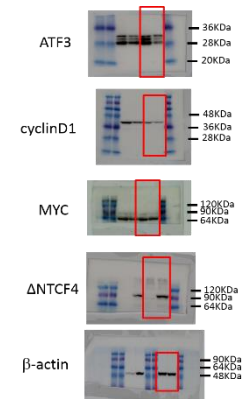

(c)

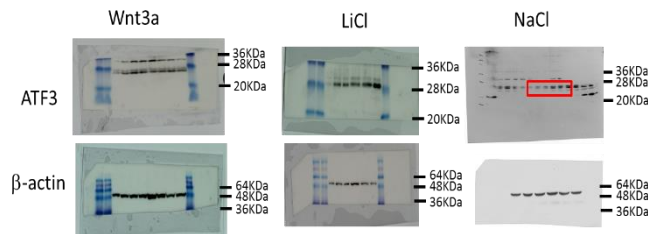

(d)

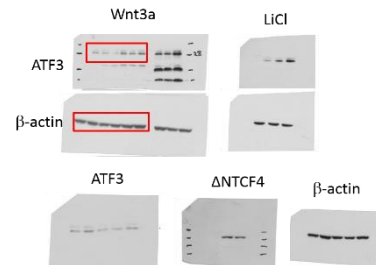

(e)

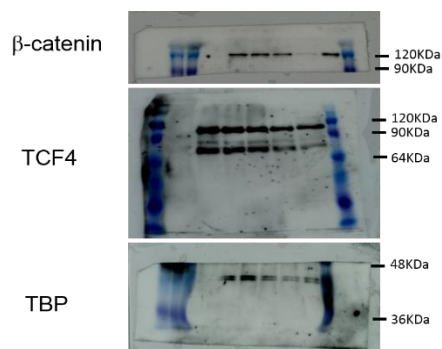

(f)

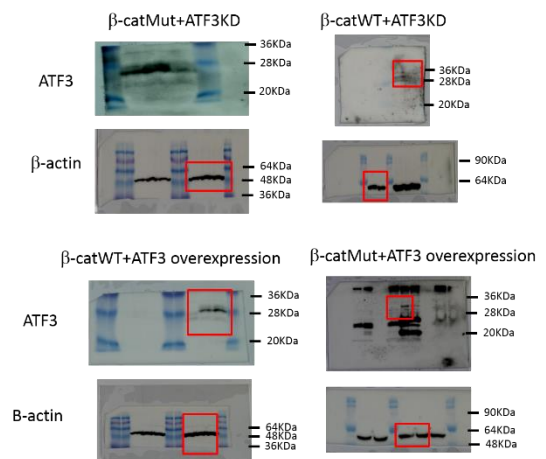

(g)

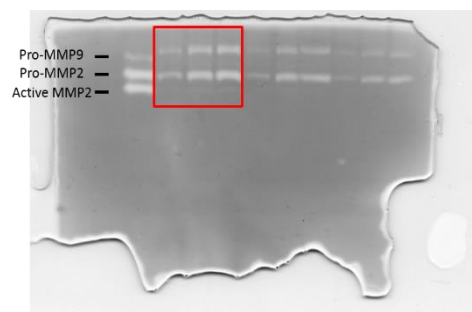

Supplement: S1 File — (a) and (b) are blots of Fig 1A and 1D, respectively. (c) and (d) are blots of Fig 2A and 2B, respectively. (e) represents blots and DNAP assay of Fig 3H, and (f) are blots of Fig 4A. (g) MMP assay gel shown in Fig 5D. (PDF) [file pone.0194160.s005.pdf]

(a)

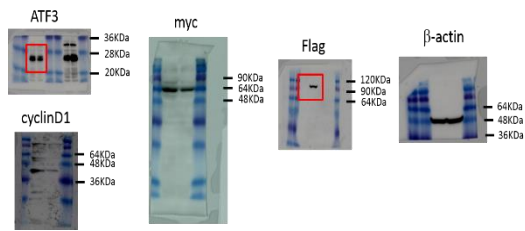

(b)

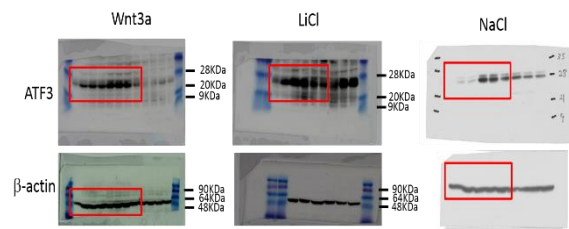

(c)

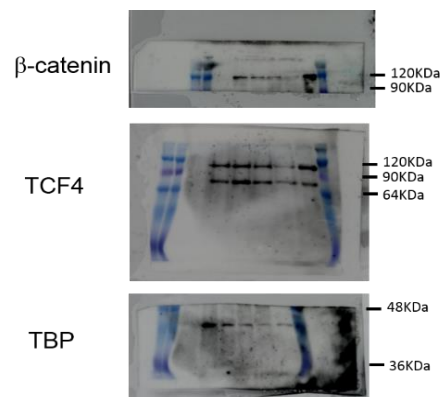

(d)

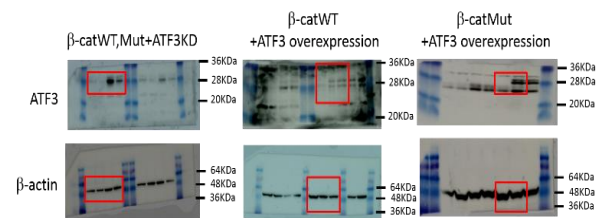

Supplement: S2 File — (a) and (b) are blots of S2B and S2C Fig, respectively. (c) represents blots and DNAP assay of S3C Fig. (d) is blots of S4A Fig. (PDF) [file pone.0194160.s006.pdf]
